# The Surgeon and Sentiment

**Published:** 1890-04

**Authors:** 


THE SURGEON AND SENTIMENT.—Under this caption the *Boston Medical and Surgical Journal* recently discussed the ideal surgeon of the laity, with the surgeon of reality, as evinced through professional and private life. The *Journal* assumes that the former is : " A man of given determination without any of those delicate refinements which might lead him to drop the tear of pity over the sorrows of the trembling victims of his scientific power. Stern judgment should belong to him as much as valor to the soldier, justice to the jurist, or sensitiveness to the poet. As the warrior revels in warfare, the lawyer in litigation, the clergyman in theology, so the surgeon should rejoice in bloody, physical calamity; it should be the breath of his nostrils, his reason for existence." As a matter of fact, we believe, says *The Medical Age*, these ideas are confined chiefly to the vulgar, and even among such, are held in the main toward those members of the profession who are most charlatanistic and ignorant. We recall three so-called surgeons who perfectly fulfilled this ideal : one whose medical knowledge was obtained solely as a hospital steward in the regular army; one a man of mediocre medical education, by nature cruel, and who really delighted in the knife and in the sight of blood, and subsequently ended his days in an asylum; the third very like the preceding, and who, though oft boasting his nerve, and delight in operating, ultimately took his own life rather than submit to the knife that would have relieved him of an otherwise incurable malady.

